# On the Remedial Efficacy of Ox-Gall

**Published:** 1845-09

**Authors:** 


*On the Remedial Efficacy of Ox-Gall.*—Dr. Allnatt of London, in a paper under this title, in a recent number of the *Lancet*, brings forward additional testimony to the beneficial effects of ox-gall in cases of constipation of the bowels.

Habitual constipation (Dr. Allnatt observes) in persons of sedentary habits, generally arises from a deficiency of bile, the motions are clay-colored, the more fluid parts become absorbed, and scybala are impacted in the large intestines. When portions of scybala removed from the body, are subjected to the action of ox-gall, they become immediately broken down and dissolved, and this effect follows when diluted ox-gall is used in the form of enema. Two cases are related in which this remedy was employed in this way:—

"A young lady, aged 20, suffered from obstinate constipation, which had persisted upwards of a fortnight. She had been treated previously by drastic purgatives, which produced pain and vomiting, and a feeling of general uneasiness, combined with ineffectual attempts to pass an evacuation. The lower portions of the intestines were evidently obstructed by impacted scybala. Injections containing turpentine were first administered without affording relief. Two ounces of ox-gall, with about half a pint of thin gruel, were next thrown into the rectum; the exterior parts of the hard mass became immediately dissolved, and in the course of ten or fifteen minutes the whole was ejected, to the instantaneous relief of the symptoms."

"A lady, aged 77, living in the country, to whom the author was hastily summoned, was apparently sinking from the effects of unrelieved constipation. Excrementitious vomiting had taken place, and the powers of life seemed waning. The question was, whether or not, from the violence of the inverted action, of the intestines, intussusception had occurred. On examination, I thought I could detect a hardened mass impacted about the head of the colon, and evidences of accumulation below that point. I therefore advised, as a last resort, an enema of ox-gall and turpentine (the latter more as a stimulant to the inactive bowel than for any specific effect) with thin gruel, to be vigorously injected, warmed, and as far as possible into the intestine. In less than half an hour a mass of scybala was expelled, the exterior of which had been imperfectly softened by the action of the gall, covered with a coating of thick mucus. Other portions speedily followed, and convalescence ensued."

In the form of pill, in the dose of five grains three times a day, the ox-gall acts with almost specific certainty (acording to Dr. Allnatt) in cases of habitual constipation, accompanied by indigestion, clay-colored stools, and a feeling of oppression after food. It acts by supplying the natural stimulus to the intestines, and an advantage it possesses is its perfectly harmless nature. It does not, however, appear to be so well suited to constipation depending upon other causes, and when the liver begins to assume its healthy action the ox-gall must be discontinued, as it will then produce all the symptons of regurgitation of bile into the stomach.

Dr. Allnatt lastly alludes to another point connected with the administration of the ox-gall, which, if borne out by subsequent experience, will render it a still more useful medicine—viz., its power of destroying the narcotizing property of opium when combined with it. "The constipating effect of opium, Dr. Allnatt observes, is principally produced by its action upon the liver, the secretion of which it arrests and renders insufficient for the due stimulation of the alimentary canal. In many cases this is a serious drawback to the exhibition of opium, for we often require its sedative when its constipating effects would be sufficiently injurious to preclude its use. Five or eight grains of inspissated ox-gall will neutralize the effect of a grain of opium without destroying its sedative efficacy; it also prevents in a great measure its injurious action upon the brain."—*Dublin Medical Press.*

